# Fever of Unknown Origin: A Case Report of Hepatic Phlegmon in an Immunocompetent Patient

**DOI:** 10.7759/cureus.59229

**Published:** 2024-04-28

**Authors:** Sandra E Pruitt, Jacob Filipek, Dustin Williford, Sara Sanders, Brittany Slagle, Heather Young, Jessica Snowden

**Affiliations:** 1 Internal Medicine, University of Arkansas for Medical Sciences, Little Rock, USA; 2 General Pediatrics/Hospital Medicine, University of Arkansas for Medical Sciences, Little Rock, USA; 3 Pediatrics/Hospital Medicine, University of Arkansas for Medical Sciences, Little Rock, USA; 4 Pediatrics/Infectious Disease, University of Arkansas for Medical Sciences, Little Rock, USA

**Keywords:** liver, abscess, phlegmon, immunocompetent, mrsa, hepatic abscess, hepatic phlegmon

## Abstract

Methicillin-resistant *Staphylococcus aureus* (MRSA) hepatic phlegmon is a rare cause of fever of unknown origin (FUO) in an immunocompetent patient from a high-income country (HIC). MRSA hepatic phlegmon is typically linked to protein malnutrition and chronic gastrointestinal infections in low- to middle-income countries while immunodeficiencies such as chronic granulomatous disease (CGD) are a more common cause in a HIC. Clinical manifestations of hepatic phlegmon can be vague and nonspecific making a complete FUO workup critical during evaluation. We report a case of MRSA hepatic phlegmon in an immunocompetent patient with a nonspecific history and physical exam findings. A 14-year-old male presented with an 11-day history of fever with mild bilateral upper quadrant abdominal pain. The patient also has mild upper quadrant pain with palpation. The patient was diagnosed with a hepatic phlegmon on abdominal ultrasound and computed tomography (CT) of the abdomen. He was started on antibiotics and Interventional Radiology placed drains into the phlegmon and performed vancomycin drain washes. Inflammatory markers were initially elevated and trended down with interventions. The patient did well with treatment and was back to baseline during outpatient follow-up with the Infectious Disease team. This case illustrates the importance of a complete workup in patients with FUO.

## Introduction

Methicillin-resistant *Staphylococcus aureus* (MRSA) hepatic phlegmon is a rare cause of fever of unknown origin (FUO) in an immunocompetent patient from a high-income country (HIC) [1]. MRSA hepatic phlegmon is more typically linked to protein malnutrition and chronic gastrointestinal infections in low- to middle-income countries (LMICs) while immunodeficiencies such as chronic granulomatous disease (CGD) are a more common cause in HICs. Similar to the local collection of suppurative material in an abscess, phlegmons are acute unconfined infections that spread along tissue planes [2]. Typical features consistent with hepatic abscess include fever, abdominal pain, and tender hepatomegaly with less typical features including weight loss, fatigue, and right upper quadrant pain [1,3].

While the true incidence of hepatic phlegmons in children is unknown due to minimal literature, the incidence of pediatric liver abscesses in the United States is approximately 25/100,000 and is much higher in LMICs [1,3-5]. The median age at initial presentation is approximately 10 years in HICs [4,6]; however, initial presentations as young as a few months of age have been reported [6,7]. Clinical manifestations of hepatic phlegmon can be vague and nonspecific making a complete and expedited FUO workup critical during evaluation as hepatic abscess has been linked to high mortality.

## Case presentation

A 14-year-old male, with a history of depression and attention deficit hyperactivity disorder, presented to an emergency department in the southeast United States with 11 days of daily fevers and mild bilateral upper quadrant abdominal pain. He was diagnosed with a presumed tick-borne illness by his primary care physician three days prior to presentation due to his recent time at summer camp in a tick-endemic area of the southeast United States and prescribed five days of doxycycline. The patient defervesced with antipyretic use but fevers continued to occur daily with maximum temperatures of 38.8°C. Associated symptoms included decreased appetite, malaise, intermittent nausea, vomiting, and unintentional weight loss of four kilograms. He denied sweats, rash, diarrhea, constipation, myalgias, and joint pain. Home medications included sertraline. He denied any recent sick contacts. He had traveled to Guatemala four months prior to the onset of symptoms. The patient’s animal contact included his Yorkshire terrier and his neighbor’s horses and goats. He denied any history of trauma. 

On physical exam, weight was 44.9 kg, temperature was 38.3^o^C, heart rate was 95 beats/minute, respiratory rate was 20 breaths/minute, oxygen saturation was 100%, and blood pressure was 108/64 mm-Hg. He was in no acute distress with very slight upper quadrant abdominal tenderness to palpation. Otherwise, skin, head and neck, cardiac, pulmonary, abdominal, extremity, and neurologic exams were normal.

Emergency department laboratory results showed leukocytosis of 18.4 K/uL with 66% neutrophils, thrombocytosis of 620 K/uL, anemia with hemoglobin of 11.5 g/dL, C-reactive protein (CRP) of 147.9 mg/L (reference range 0.0-10.0 mg/L), and erythrocyte sedimentation rate (ESR) of 51 mm/hour. He had a normal complete metabolic profile including sodium 137 mmol/L, potassium 4.4 mmol/L, chloride 98 mmol/L, bicarbonate 25 mmol/L, blood urea nitrogen 10 mg/dL, creatinine 0.4 mg/dL, glucose 92 mg/dL, aspartate aminotransferase (AST) and alanine transaminase (ALT) were both 22 U/L. Lactate dehydrogenase (LDH) and uric acid were normal. HIV and monospot tests were negative. He was admitted for FUO.

**Figure 1:**
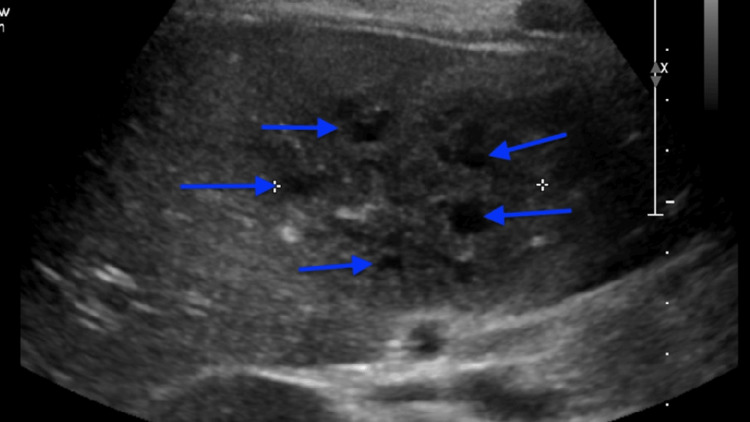
Abdominal ultrasound of liver phlegmon in transverse view, centrally showing multiple hypoechoic phlegmons without coalescence to abscess as denoted by blue arrows

Additional ED infectious workup included blood culture, HIV, and cytomegalovirus (CMV)/Epstein-Barr virus (EBV), all of which were negative. The patient was empirically started on metronidazole, vancomycin, and ceftriaxone while remaining on doxycycline. He continued to experience decreased appetite and periodic right upper quadrant pain without discernible associations. An abdominal ultrasound showed multiple hypoechoic lesions in the right lobe of the liver as seen in Figure 1. Computerized tomography (CT) of the abdomen and pelvis also showed these lesions. The radiology report stated that there were multiple adjacent hypoechoic lesions in the right lobe of the liver, most consistent with hepatic phlegmon without coalescence to abscess. There were no other significant findings.

After admission and discussion with the Infectious Disease team, concerns for immunodeficiency workup were raised. Additional testing including immunoglobulins (A, E, G, and M were obtained, D was not) was normal, neutrophil oxidative burst testing showed appropriate response to stimulation with the fluorescence of dihydrorhodamine indicating negative chronic granulomatous disease (CGD). The patient was fully vaccinated though vaccine titers were not sent. Based on these results, the patient was considered immunocompetent. On Hospital Day 2, Interventional Radiology aspirated 3 mLs of fluid from one of the lesions. On Hospital Day 6, the phlegmon aspirate grew MRSA. Table 1 shows the speciation and susceptibilities.

**Table 1:** Antibiotic susceptibilities to MRSA grown from culture of hepatic phlegmon sample MIC: minimum inhibitory concentration; MRSA: methicillin-resistant *Staphylococcus aureus*

Antibiotic	*Staphylococcus aureus* susceptibility, MIC in ug/mL
Clindamycin	≤ 0.5 ug/mL Susceptible
Daptomycin	≤ 1 ug/mL Susceptible
Oxacillin	>2 ug/mL Resistant
Trimethoprim + sulfamethoxazole	≤ 0.5/9.5 ug/mL Susceptible
Vancomycin	1.5 ug/ml Susceptible

Ceftriaxone and metronidazole were discontinued. Clindamycin was added to vancomycin treatment due to poor response to monotherapy and literature for adult patients showing positive outcomes on the use of combination therapy for the treatment of MRSA abscess. Repeat liver ultrasound on Hospital Day 10 showed a slight decrease in the phlegmon’s size. Interventional Radiology and Surgery were again consulted as the patient continued to spike fevers despite aggressive antibiotic coverage and to reassess potential surgical options, and both agreed no surgical intervention was warranted unless the phlegmon coalesced. Beginning on day 20, the patient’s fever curve steadily worsened with increasing frequency of fever spikes and maximum temperature values up to 40^o^C. Another abdominal ultrasound on Hospital Day 23 again showed no coalescence or any other significant changes. At this point, Pediatric Infectious Disease reached out for consultation with local Adult Infectious Disease for additional expertise given the unique presentation of our patient and poor response to current antibiotics. Daptomycin was started on day 25 while vancomycin was stopped, and clindamycin continued. The fever curve did not respond to daptomycin with a maximum temperature of 40^o^C and daily fevers continued. Our infectious disease specialists felt that if there was a possibility of drain placement within areas of the phlegmon, the patient would respond better to antibiotic use.

**Figure 2:**
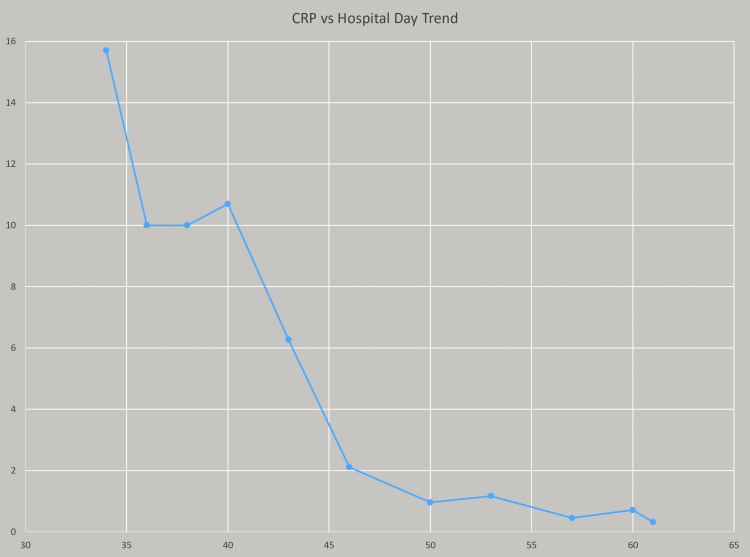
Graph showing hospital day on the X-axis and CRP trend on Y-axis measured as mg/dL; on hospital day 40, vancomycin flushes via hepatic drains and IV piperacillin/tazobactam were added to treatment regimen. CRP: C-reactive protein

The patient was transferred on day 30 to a facility with a pediatric liver surgeon due to a high risk of surgical complications, particularly encroachment of the hepatic vasculature. At the quaternary facility, the interventional radiology and liver specialist team placed three drains within the phlegmon using ultrasound guidance and CT scans of the phlegmon from their facility. The patient was started on linezolid and rifampin at the quaternary facility based on their infectious disease specialists' preference and in the setting of poor response to combination vancomycin, clindamycin, and daptomycin therapy at our facility. The patient continued to have fever spikes, and on Hospital Day 40, vancomycin flushes via drains placed by the interventional radiology team were added to the patient's treatment regimen. On Hospital Day 40, the quaternary facility also started piperacillin/tazobactam in the setting of persistent fevers and concern for potential polymicrobial infection though no other microbes were identified from hepatic phlegmon or blood cultures except MRSA. At this point, the patient responded slowly to treatment with a downtrend in his CRP. Table 2 and Figure 2, show the CRP trend with the timeline including drain placement on Hospital Day 34, drains flushed by Interventional Radiology with vancomycin, and piperacillin/tazobactam started on Hospital Day 40.

**Table 2:** CRP trends by hospital day CRP: C-reactive protein

Hospital Day	34	36	38	40	43	46	50	53	57	60	61
CRP milligram/deciliter (mg/dL); Normal value < 0.8 mg/dL	15.7 mg/dL	10 mg/dL	10 mg/dL	10.7 mg/dL	6.27 mg/dL	2.11 mg/dL	0.96 mg/dL	1.17 mg/dL	0.45 mg/dL	0.71 mg/dL	0.322 mg/dL

The patient was discharged home after four additional weeks of inpatient therapy at the quaternary facility. He was transitioned to oral clindamycin before discharge and followed up in the Pediatric Infectious Disease clinic at our facility. At the follow-up visit, labs were normal including hemoglobin and CRP; the patient's appetite had improved with appropriate weight gain following recovery and resolution of other presenting symptoms. Follow-up CT imaging of the liver was done at our institution due to the complex nature of the case, showing resolution of hepatic phlegmon.

## Discussion

*Staphylococcus aureus* is the most common isolated pathogen worldwide from pediatric liver abscesses followed by *Entamoeba histolytica* [1,8,9]. In the literature review, only one case of a pediatric MRSA hepatic abscess was found in a HIC without risk factors (i.e. CGD) similar to our current case [10]. Of the approximately 20 other cases documented, most were from Brazil and Argentina with a few cases of neonatal hepatic abscesses [9-12], predominantly amoebic in etiology.

Fever is the universal symptom of a hepatic abscess. Abdominal pain and hepatomegaly are common but not universal and their absence should not exclude hepatic abscess from the differential diagnosis [12-16]. In fact, previous case studies have found only half of all children who were later diagnosed with a hepatic abscess presented with abdominal pain initially [4,6]. A single abscess can present slowly with nonspecific symptoms such as weight loss, fatigue, and right upper quadrant pain while multiple abscesses usually present more acutely with more notable abdominal pain and weight loss [1]. Other less common presentations include nausea and vomiting, cough, and respiratory distress. Almost all cases present with leukocytosis, normocytic anemia [16], and elevated inflammatory markers while liver enzymes are elevated in less than 50% of cases [4,5]. One study has also shown roughly 30-39% of patients with hepatic abscesses also have concurrent bacteremia [17]. In another study, 80-90% of cases also had prolonged prothrombin time though no clear rationale for this finding is known [12]. Patients who present with hepatic abscess or phlegmon without a history of blunt trauma or source of intra-abdominal infection should have immunologic workup to assess for immunodeficiencies, with follow-up based on results of testing [6].

Ultrasound is the imaging study of choice for the initial diagnosis of hepatic abscesses [5]. CT is more sensitive for detecting hepatic abscesses and can also be used to assess for other abscesses and sources of infection [5,6]. However, due to the risk of contrast nephropathy and radiation exposure, ultrasound should be used for all follow-up imaging, unless a CT scan is warranted due to distinct needs [18].

The initial antibiotic regimen should cover the most common organisms responsible including *Staphylococcus aureus*, gram-negative bacteria, and anaerobes. A suggested empiric regimen includes vancomycin, a third-generation cephalosporin, and metronidazole [13]. Most patients will require some form of surgical drainage in addition to medical treatment [13-15]. Antibiotics along with percutaneous aspiration are considered first-line treatment in most cases of unilocular liver abscesses [1]. Open drainage may be indicated if the liver abscess is multiloculated, has multiple abscesses, and is close to the pleura, or the patient has coexistent conditions requiring surgery [14,16]. Antibiotics should be continued for four to six weeks after adequate drainage [1].

In the current case, we initially started treatment broadly then narrowed treatment to only vancomycin based on the Infectious Diseases Society of America (IDSA) guidelines. There are some recent concerns that there is slow bactericidal activity, emergence of resistance, and increasing minimum inhibitory concentrations (MICs) in susceptible strains [19]. As treatment response did not go as expected, we then broadened coverage to include vancomycin and clindamycin based on data suggesting that combination therapy could improve response to treatment [20]. Vancomycin is known to work well for MRSA but has shown less efficacy once MIC >2 [20]. Other treatment modalities include daptomycin though it should be considered after vancomycin treatment failure as daptomycin’s pharmacokinetics and efficacy are under investigation in the pediatric population [20]. Additional combination options include linezolid and rifampin, as linezolid is bacteriostatic against staphylococcal infection by inhibiting protein synthesis at the 50S ribosome, and rifampin is a good adjunctive therapy as it has bactericidal activity and achieves high intracellular penetration but with high resistance rates as monotherapy [20]. Intra-abscess antibiotic injection has also shown success as part of the treatment plan [21], and was employed during the treatment of the patient discussed. In the treatment of hepatic abscess, consideration can be taken of polymicrobial infection and broadening in particular for anaerobes and other GI tract bacteria; in our case, the patient had improved response once piperacillin/tazobactam was started for broadened coverage in concert with other treatment modalities.

Pediatricians should recognize the need to refer patients to specialized care when they are not responding to antibiotics and percutaneous aspiration as expected. Due to the rarity of pediatric liver abscesses, pediatric surgical and interventional radiology teams may lack the experience needed to safely perform percutaneous drainage and open surgical techniques. Since the mortality remains high if this disease process is left untreated, the decision to transfer should be made quickly when conservative medical management has been exhausted [1,22]. In our case, we transferred our patient to a facility with a pediatric hepatic surgical specialist.

## Conclusions

MRSA is an uncommon culprit in an uncommon diagnosis of pediatric hepatic phlegmon in a previously healthy immunocompetent adolescent. This case illustrates that hepatic abscesses can cause mild to minimal abdominal discomfort on presentation and that many lab values related to liver injury such as transaminases and LDH can be deceptively normal. For patients who do not improve in 48-72 hours with appropriate antibiotics and whose phlegmons or abscesses are unable to be aspirated, we suggest immediate transfer to an institution with experienced pediatric hepatic surgeons to help guide the treatment plan.

Waiting for coalescence of a hepatic phlegmon may delay treatment and improvement for the patient, and we believe that transfer to a facility with a pediatric hepatic surgical specialist will help expedite treatment and recovery. Our patient had a hepatic phlegmon requiring a specialist for adequate drainage but was responsive to expected management post drainage. This case emphasizes the importance of transferring to a surgical specialist when more conservative medical management modalities have been exhausted.
